# Review Department

**Published:** 1890-02

**Authors:** 


### REVIEW DEPARTMENT.

THE EXTERMINATION OF THE AMERICAN BISON. By WILLIAM T. HORNADAY, Superintendent of the National Zoological Park. 8vo, plates and map. Washington, D. C. 1889.

Some time ago Congress voted $200,000 for the establishment of a National Zoological Park in the District of Columbia, the main purpose of which was for the preservation of specimens of every species of American quadruped now threatened with extermination. To prove the wisdom of this determination and the necessity of prompt action in the matter would seem to be a very good *raison d'etre* for Mr. Hornaday's publication of this report.

It contains an exhaustive account of the habits and haunts of the far-famed buffalo, or, more properly speaking, bison, and the sad story of its almost entire destruction. In 1870, 20,000,000 of these magnificent animals roamed the plains; to-day there are less than 500 wild bisons in existence, most of them killed for the sake of their hides, worth no more than a dollar apiece. The few that remain are in danger of utter extinction by crossings and in-breeding. Moreover, experiments in breeding bison bulls to domestic cows have been very successful and are now going on to such an extent that it is fair to state that in a few years the genuine bison will cease to exist. The necessity of immediate steps being taken for the protection and care of the few hundred now in the Yellowstone Park is strongly urged. The uncertainty of the fate of the park is pointed out, whilst the danger of pressure from railway corporations, which seek possession of it, causing it to be eventually broken up, is dwelt upon. The result of such a course would be undoubtedly the destruction of the entire herd.

The book is divided into three parts. The first gives the history of the bison, geographical distribution, etc. The second contains a graphic account of the cause of its extermination, and the third is the relation of the efforts of an exploring party fiom the Smithsonian Institution to obtain specimens for the National Museum. The book contains a number of fine illustrations and a map illustrating the "exterminating of the American bison."

W. A. C.

THE AGRICULTURAL GRASSES AND FORAGE PLANTS OF THE U. S., AND SUCH FOREIGN KINDS AS HAVE BEEN INTRODUCED. By DR. GEORGE VASEY, BOTANIST. U. S. Department of Agriculture (Botanical Division), Special Bulletin, 1889.

A general description of the grasses of the United States, and the soils to which they are adapted, precedes a special description of the botanical classification, vulgar names and habitat. An appendix, by Clifford Richardson, gives an analysis of the chemical composition of them at various periods of their maturity and when grown on richer or poorer soils. One hundred and fourteen plates greatly enhance the value of the work.

